# Mr. Prescott Hewett’s Lectures

**Published:** 1855-08

**Authors:** 


Mr. Prescott Hewett's Lectures.—Mr. Prescott Hewett terminated his course of lectures at the College of Surgeons on the 12th instant, by a recapitulation of seven various forms in which effusion of blood, as previously described, are found in injuries of the head. In thirty-one cases of injury with sanguineous effusion, twenty-seven were found to be in the anterior part of the head, and four in the posterior or cerebellar portion. The lecturer dwelt on the various differential signs of compression and concussion, more particularly the instantaneous quickness of the phenomena of concussion, while a particular interval must elapse, as in many patients walking to hospital themselves, with compression or even effusion of blood, some hours after the injury, blood all the time, drop by drop it might be, making its way into the brain. Le Dran, Pott, and other surgeons, it was observed, dwelt particularly on the value of the interval, as a direction to the surgon whether he was to use the trephine or not. In practice, in twenty-five cases at St. George's Hospital, for instance, the brain itself was found by Mr. P. Hewett to be lacerated; so here the trephine was not of any use; while, in a large number of cases, compression and concussion are so intimately connected that it is almost impossible for the surgeon to disentangle the thread of symptoms. If paralysis be present, it is best to trephine at once, of course at the opposite side. Professor Hewett gave a striking case of a man, who received an injury at two o'clock in the day, drove himself six miles after the accident, and fell insensible at six o'clock, on reaching St. George's. There was apparently no fracture, no paralysis, and the man died next morning, though every effort was made to find out the seat of injury. After death the dura mater was found separated from the bone by a large effusion of blood. This was the type of a vast number of cases. Extravasation into the arachnoid is, however, the most common; this it is important to remember. The lecturer dwelt at unusual length on the vascularity of clots of blood in the brain, as shown by Paget, Gray, and others. He felt convinced that these changes explain the origin of cysts in the brain. In a most singular case given by Dr. R. Quain, in which a man had injury of the brain, but died some long time after of fatty heart, and who was subject to fits, the latter were explained by such cysts. On a post-mortem examination in this case a large cyst rolled out on the table. Effusion of blood into the pia mater was less frequent; these and effusions into the brain may be the cause, as in apoplexy, of surgical accidents, not the consequence. In the previous lecture (the fifth) Mr. Hewett dwelt chiefly on the surgical treatment of concussion and compression, or rather on those points still open to doubt and the teachings of modern experience; pyæmia also was considered. Inflammation of the dura mater, according to Mr. Guthrie and others, it was observed was unknown. Two hundred cases of Dr. Pott were cited, with only five deaths; these were of very doubtful value. The absence of hospital records in London hospitals was, he thought, very much to be deplored. Bloodletting, according to Pott, was very useful in concussion, but modern surgery was opposed to this. He (the lecturer) thought we did not discriminate enough; he would, individually, go *in medio.* In one class of cases it was of no use; in another it was imperatively called for. Pott saw symptoms of effusion under the bone removed by bleeding; acute inflammation also was not uncommon in the sub-arachnoid tissues. The lecturer gave an instance from St. George's: wound of scalp; bone dry, etc.; but twenty-nine days after, on a post-mortem, pus and lymph were found under the bone and along the middle meningeal artery; purulent infiltration also into the lungs. In another case, also of diffuse inflammation of the membranes, the man got quite insensible; there were no indications for the trephine, yet the membranes of the brain were pressed on by purulent effusion. Where we have coma and hemiplegia, the surgeon is quite justified in trying the trephine. Dupuytren had six successful cases, but the lecturer had searched in vain for them in French books. Pott had nine, in which was found pus in large quantities; *no paralysis!* In some cases Mr. Cock, of Guy's Hospital, pus was found in the arachnoid. Dupuytren used an issue experimentally to draw off the pus! The lecturer could scarcely believe the cases of Pott and Dupuytren. Again, abscess of the brain, as spoken of by some authors, was rather a circumscribed effusion of pus into the membranes, with plastic lymph. An interesting diagram was here explained, where the purulent matter made its way before it, pushing down the corpus callosum; the hemisphere itself was pushed on one side from the falx, but there was nothing which one could call an abscess. Mr. Hewett concluded this lecture by referring to casualties, such as pyæmia, or purulent infection—as he thought, a better term—a disease of which he took the most gloomy view; in fact, when the fatal rigor, sweating, haggard and sickly aspect of the hospital patient, after operation, set in, he did not know what to do. In eighteen cases of death, he found the lungs affected in thirteen, not the liver, as generally supposed. In twenty-three other cases of Mr. H. Lee's, fourteen showed purulent deposits also in the lungs; this was very important. Such cases were best treated, perhaps, medically; but a great change had been recently made by Sir B. Brodie and others—namely, to feed the patient and keep up the strength. The lecturer dwelt on the propriety of attending medically to the condition of the kidneys, as albuminuria was very common in scalp wounds. An albuminous state of the urine, at least, runs parallel with a state of constitution favorable to a low inflammatory condition of scalp wounds, pyæmia, etc. Flap wounds of the scalp, as a rule, however, rarely slough. The lecturer would not be afraid of sutures in such cases. The occipito frontalis should not be included. Wounds of arteries of the scalp require ligatures at both ends; punctures (Dobson's plan), on the other hand, were not necessary. The punctures were more valuable, where there is puffiness of integument. In erysipelas, quinine, iron, and ammonia, are our sheet-anchors, to adopt a strong phrase. Cases of Sir B. Brodie were given where the patient was dying under the cold orthodox calomel, bleeding, and antiphlogistics, but cured by this eminent surgeon ordering the very opposite—gin, wine, and other stimulants. The lecturer gave a curious case of Saviard, in the original French, and also dwelt on the profusion of holes formerly made in the skull by the old-fashioned trephines, twenty-seven crowns of a trephine, in one case, going down to the dura mater—a remedy worse than the disease. Mr. Hewett also cited cases of deafness and diseased bones of the ear, with paralysis of face, depending also on irritation or injury of the dura mater. Pott conceived this arose from separation of the internal periosteum. The lecturer also pointed out the relation of the sinuses of the mastoid and frontal bones in some of the lower animals. In the elephant these sinuses extended to ten inches and a half of diploe of bone—a wise provision of Nature to defend the brain of the lower animals from external accidents.—*London Lancet.*

